# Human cystic echinococcosis in Heilongjiang Province, China: a retrospective study

**DOI:** 10.1186/s12876-015-0256-8

**Published:** 2015-03-10

**Authors:** Tiemin Zhang, Wei Zhao, Dong Yang, Daxun Piao, Shibo Huang, Yuanyuan Mi, Xianqi Zhao, Jianping Cao, Yujuan Shen, Weizhe Zhang, Aiqin Liu

**Affiliations:** 1 Department of General Surgery, The First Affiliated Hospital of Harbin Medical University, Harbin, Heilongjiang 150001 China; 2 Department of Parasitology, Harbin Medical University, Harbin, Heilongjiang 150081 China; 3 National Institute of Parasitic Diseases, Chinese Center for Disease Control and Prevention, Key Laboratory of Parasite and Vector Biology, Ministry of Health, WHO Collaborating Centre for Malaria, Schistosomiasis and Filariasis, Shanghai, 200025 China

**Keywords:** Cystic echinococcosis, Retrospective analysis, Humans

## Abstract

**Background:**

Cystic echinococcosis (CE) is one of emerging zoonotic parasitic diseases throughout the world, having significant medical and economic importance in developing countries. The western and northwestern China is considered as CE endemic areas. In northeastern China’s Heilongjiang Province, the increasing number of sporadic human CE cases has attracted more and more attention. The aims of the present study were to understand the clinical characteristics of human CE in the investigated area and to compare the coincidence rates of CT, ultrasound and serological test against the histopathology results among CE patients.

**Methods:**

Hospital data of 183 human CE cases in the period from January 2004 to July 2013 were collected from the two largest hospitals in Heilongjiang Province. Clinical data were analyzed, including age, gender, occupation and living residence of CE patients and localization, size and number of CE cysts as well as the diagnosis methods of CE before operation.

**Results:**

The results revealed that the incidence of CE reached a peak in the age group of 41–50 years. Among the 183 CE patients, the females were observed to have a higher percentage of CE patients (60.66%, 111/183) than males (39.34%, 72/183). The majority of CE patients were farmers, followed by workers, employees, public servants, students and so on. CE cysts were most commonly found in the livers, with a 30 cm cyst in diameter being detected. CT showed the highest coincidence rate (96.64%) for hepatic CE among the three common diagnosis methods (CT, ultrasound imagine and serological test) compared against the histopathology results.

**Conclusions:**

This is the first retrospective analysis of human CE cases in Heilongjiang Province in recent ten years. Clinical characteristics of human CE were described here. CT appeared to be the most effective diagnosis method for hepatic CE.

## Background

Cystic echinococcosis (CE), caused by *Echinococcus granulosus* in larval stage, is considered as one of the most dangerous zoonotic parasitic disease worldwide, mainly distributing in Mediterranean regions, Russia, central Asia, China, Australia, South America, and north and east Africa [1]. Clinical manifestations of human CE vary from asymptomatic infection to severe morbidity and mortality depending on the size and localization of the cysts, complications and the host’s health status [2]. CE not only shows public health importance but also has caused economic problem [3]. CE is principally maintained in a dog–sheep–dog cycle. Humans are an accidental intermediate host for this parasite, and normally infected by ingestion of eggs released from dogs or other canids. The larvae emerging from the eggs give rise to hydatid cysts, which are mostly found in the livers of hosts.

Currently, China has been listed as one of the most important endemic regions of echinococcosis [1]. At least 35,000 human CE cases have been treated surgically in the last century since1950s [4], distributing in 27 provinces, autonomous regions, and municipalities, with western and northwestern China being the main endemic areas [5,6]. Heilongjiang Province is in the northeast of China. Since the first human CE case was reported in 1958, the number of sporadic CE patients in hospitals has been increasing, especially in recent years [5]. In the present study, we collected and analyzed the clinical data of human CE patients, who were subjected to surgical operation in the two largest hospitals in Heilongjiang Province. The aims of this retrospective analysis were to understand the clinical characteristics of human CE in Heilongjiang Province, and to compare the coincidence rates of computed tomography (CT), ultrasound and serological test against the results of histopathology among CE patients.

## Methods

### Study location and screening of human CE cases

The data of human CE cases were collected from the first Affiliated Hospital and the Second Affiliated Hospital of Harbin Medical University, where the majority of CE patients residing in Heilongjiang Province were operated surgically. The CE patients involved in this analysis were subjected to surgical operation and confirmed by histopathology in the period from January 2004 to July 2013.

### Data collection

The age, gender, occupation and living residence (city and countryside) of CE patients were extracted from hospital records. Clinical records of the cysts were collected, including the localization, size and number of them. In addition, the data on the diagnosis methods of CE before operation were obtained, including CT, ultrasound image and serological test.

### Ethics statement

This research study was approved by the Medical Ethics Review Committee of Harbin Medical University. This was a retrospective analysis of routine clinical data and therefore we requested and were granted a waiver of individual informed consent from the ethics committee.

## Results

### The number of human CE cases by year (2004–2013)

**Figure 1:**
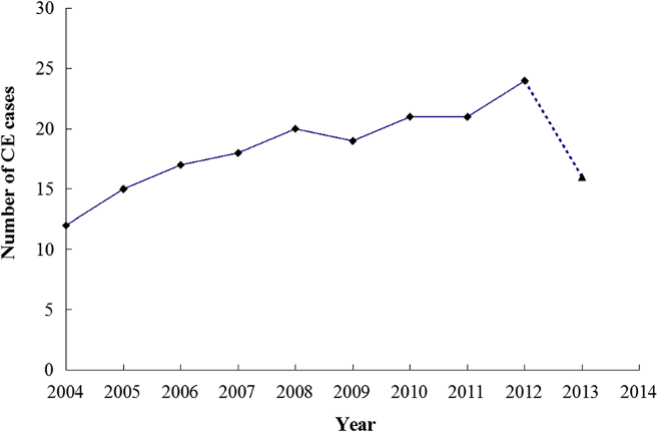
**Number of CE cases in recent 10 years in Heilongjiang Province by year. ▲**In 2013, CE cases were collected only in the first seven months.

The number of human CE cases generally showed an increasing tendency in recent years, with the smallest and largest case numbers in 2004 (n = 12) and 2012 (n = 24), respectively. 16 human CE cases were obtained from January to July in 2013 (Figure 1).

### Age, gender and occupation of human CE patients

The age and gender distributions of CE patients were summarized in Table 1. Of 183 CE patients, their ages ranged from 6–72 years with the mean age of 45.61 years. The largest share (27.32%, 50/183) of CE patients was found to be in the age group of 41–50 years. 31–40-year-old CE patients constituted the second largest share (22.95%, 42/183). Meanwhile, females (60.66%, 111/183) were more than males (39.34%, 72/183).

**Table 1:** **Age and gender distributions of CE cases**

Age (years)	Males (%)	Females (%)	Total (%)
6-10	4 (5.56)	1 (0.90)	5 (2.73)
11-20	9 (12.5)	2 (1.80)	11 (6.01)
21-30	20 (27.78)	8 (7.21)	28 (15.30)
31-40	9 (12.50)	33 (29.73)	42 (22.95)
41-50	18 (25.00)	32 (28.83)	50 ( 27.32)
51-60	8 (11.11)	21 (18.92)	29 (15.85)
61-72	4 (5.56)	14 (12.61)	18 ( 9.84)
Total	72 (39.34)	111 (60.66)	183 (100.00)

The occupations and living residences of CE patients were illustrated in Table 2. 183 CE patients were divided into six groups in our study according to their occupations: farmers (n = 83), workers (n = 37), employees (n = 17), public servants (n = 13), students (n = 11) and others (n = 22), including two doctors, two drivers, two preschool children, three teachers, three businessmen, five unemployeds and five housewives. Among them, 126 and 57 of CE patients were from countrysides and cities, respectively (Table 2).

**Table 2:** **Occupation and residence of CE patients**

Occupation	Number (%)	Residence	
		Countryside (%)	City (%)
Farmer	83 (45.36)	83 (65.87)	0 (0.0)
Worker*	37 (20.22)	19 (15.08)	18 (31.58)
Employee	17 (9.29)	2 (1.59)	15 (26.32)
Public servant	13 (7.10)	5 (3.97)	8 (14.04)
Student	11 (6.01)	8 (6.35)	3 (5.26)
Others	22 (12.02)	9 (5.73)	13 (22.81)
Total	183 (100.00)	126 (68.85%)	57 (31.15%)

*Workers referred to the people working in local factories.

### Localization, size, and number of cysts

The majority of the cysts were in livers (95.08%, 174/183), followed by 2.73% (5/183) in lungs, 1.09% (2/183) in spleens and 1.09% (2/183) in brains. The mean diameters were 7.4 cm, 5.2 cm, 13.5 cm and 4.9 cm for the hepatic, pulmonary, splenic and cerebral cysts, respectively. 139 patients had a single cyst with 131, four, two and two in livers, lungs, spleen and brain, respectively; 44 patients had at least two cysts with 43 and one in livers and lungs, respectively (Table 3).

**Table 3:** **Localization, size and number of hydatid cysts**

Localization (n)		Percentage (%)	Cysts				Mean diameter (cm) (range)
			Single	Percentage (%)	Multiple	Percentage (%)	
Liver(174)	Right lobe (95)	95.08	75	75.96	20	24.04	7.4 (1.7-30.0)
	Left lobe (70)		56		14		
	Both lobes (9)		0		9		
Lung (5)		2.73	4		1		5.2 (2.8-11)
Spleen (2)		1.09	2		0		13.5 (13.0-14.0)
Brain (2)		1.09	2		0		4.9 (3.5-6.3)

### Comparison of diagnosis methods

For hepatic CE cases, 174, 153 and 145 of them were subjected to CT, ultrasound and serological test (ELISA for IgG antibody detection) before operation, respectively. Only 119 cases were tested by all the three methods, with 96.64% (115/119) by CT, 88.24% (105/119) by ultrasound and 62.18% (74/119) by ELISA being in agreement with the results of histopathology. Among all the five pulmonary CE cases tested by CT, three were compatible with the results of histopathology with one being positive for IgG antibody. One of the two splenic CE cases was diagnosed as CE infection either by ELISA or by CT while the other was negative for IgG antibody, but suspectedly diagnosed as CE infection by CT; however, ultrasound could not give both of them a definitive diagnosis. Among the two cerebral CE cases, one was only subjected to a serological test, but had no specific IgG antibody response while the other was given a positive diagnosis of CE infection based on CT without a serological test.

## Discussion

In our retrospective analysis of human CE cases, the number was observed to be doubled in the recent ten years (Figure 1). However, the true reason for the increasing number of sporadic human CE cases is not clear. Dogs, pigs and sheep have been confirmed previously to be infected with *E. granulosus s.l.* in Heilongjiang Province [7,8]. The ecological environment might be a main factor. In the recent decade, the national and international travels and livestock trades have been increasing as well as the number of imported dogs because of the pet raising. Thus, we can not rule out the possibility that some CE patients were infected in a known endemic region of China or other countries.

In our analysis, CE patients were observed to reach a peak in the age group of 41–50 years. It was in agreement with the result in Turkey, where most of the cases appeared in 41–50 (22.68%) years [9]. A similar result has been reported in Tunisia that most of the CE cases were aged 30–44 years, followed by the second age-group ranging from 45–59 years [2]. In general, the peaks of human CE cases were not identical to one another in different studies, however, the prevalence of *E. granulusos* infection generally increased in age. The results may be associated with the fact that *E. granulosus* infections remain silent for years before the enlarging cysts cause symptoms in the affected organs although causative infection may often be acquired early in life.

In the analysis of gender distribution of CE patients in the present study, the females had a higher occurrence of CE patients (60.66%, 111/183) than males (39.34%, 72/183). 58.6% female CE patients were from the two age groups of 31–40 and 41–50 years. Similar results were observed in case analyses of human CE in Tunisia, Jordan, Iran and China [2,10-12]. The phenomenon may be related to the fact that adult females expose to the *E. granulosus*-infected dogs and the contaminated environment more frequently than adult males due to home activities, such as feeding dogs and milking livestock. However, analyses of surgical cases of human CE in Kyrgystan and Turkey drew an opposite conclusion, and Torgerson et al. pointed out that men were more likely to seek treatment once clinical signs presented based on the fact of no gender bias in CE cases detected in the ultrasound study [9,13]. In Jordan, males younger than 15 years of age showed significantly higher surgical incidence of CE cases than females of comparable age; whereas the number of female cases of different age groups over 15 years of age was consistently higher than that of males [12]. The reason for distribution difference of CE in age and gender is unclear. It may be related to the virulence of different genotypes of *E. granulosus*, the infective dose of the parasite and health status of hosts.

In our analysis, 45.36% (83/183) of CE patients were farmers. In the rural areas of Heilongjiang Province, with the development of modern agriculture, especially in recent years, some farmers were liberated from their complex manual work and began to keep the economic animals, such as cattle, sheep and pigs. Meanwhile, domestic dogs are kept in large numbers by local farmers to guard property and livestock. The presence of dogs and sheep in the same area provides the possibility to complete the life cycle of *E. granulosus*. In the epidemiology of human CE, dogs are generally considered to be the major definitive host transmitting *E. granulosus* to humans. The poor hygiene practices also increase the opportunity of dogs and sheep infected with the parasite, respectively through the feeding of infected offal from sheep and eating food and drinking water contaminated by eggs from dogs. The farmers have opportunity to be infected with the eggs of *E. granulosus* by farming activities involving livestock and dogs, and/or by home slaughtering practices. Meanwhile, local fur industry might lead to CE infection of workers. In the present analysis, the number of CE cases was found to be larger in countrysides than in cities. This might result from the different living environments of urban and rural inhabitants.

Hydatid cysts of *E. granulosus* are the most common in livers and lungs although they can be found in any part of the human body. In the present analysis, the livers were also found to be the most affected organ (95.08%, 174/183). The result was similar to previous findings that the majority of cases presented as isolated liver disease [14-16]. However, with an exception in South Africa, the lungs were the most commonly affected organs, accounting for 11 of the 14 CE patients having pulmonary CE alone and the remaining three having pulmonary and abdominal CE; meanwhile, it was pointed out that the strain of *E. granulosus* causing disease, genetic predisposition or co-infections with HIV and/or TB might play a role [17]. The report of more pulmonary cases in children might be attributable to a lower tolerance of hydatid cyst development in pulmonary tissue in children, and thus they were more likely to present for treatment [13].

Variability in measurements of cyst size has been documented in human CE cases. Our retrospective analysis revealed that the mean diameters were 7.4 cm, 5.2 cm, 13.5 cm and 4.9 cm for the hepatic, pulmonary, splenic and cerebral cysts, respectively. The largest cyst was from a case of hepatic CE with 30 cm in diameter. In Tunisia, the mean diameter of the hepatic cysts was 8.3 cm [2]. In Libya, the diameter of the hepatic cyst was high up to 40 cm [18]. Intra-abdominal CE cyst localization was found to be related to bigger cyst size by analyzing clinical data from 91 CE patients in Turkey [19]. However, a study of CE in slaughtered animals showed that the cysts in the lungs was larger than those in the livers [20]. So far, there has been no reasonable explanation about why CE cysts appeared markedly different in size. Whether they are related to the affected organs is unclear besides the living time of cysts in hosts.

In our analysis, occurrences of CE patients with a single cyst and multiple cysts were 75.96% (139/183) and 24.04% (44/183), respectively. Similarly, in Tunisia, 70.7% (29/41) of hepatic CE patients had a single cyst, and 29.3% (12/41) had multiple cysts [2]; in Kyrgystan, a single cyst was found in 83.57% (529/633) of CE patients while multiple cysts in 16.43% (104/633) [13]. In general, CE patients with a single cyst are more than those with multiple cysts.

The coincidence rates of the three methods were tried to compare against the results of histopathology in 119 hepatic CE cases. CT showed the largest coincidence rate (96.64%) than ultrasound and ELSIA for hepatic CE patients. Two previous studies described that a positive diagnosis was obtained before operation in 97.11% (34/35) and 90% (27/30) hepatic CE patients based on Ct and ultrasound, respectively [21,22]. The results above might be related to the fact that CT gives more accurate description of the cyst characteristics compared to ultrasound as well as the cyst location. Suwan found that sonography was superior to CT in the characterization of cyst content, but CT was superior to sonography in detecting gas within the cysts and minute calcifications [23,24]. Ultrasound is gradually used in the epidemiology of human CE because of its convenience and it is often combined with serological test to diagnose CE. In Libya, 69% (233/339) out of the ultrasound-positive CE cases were antibody seropositive while 11.2% (12/106) out of ultrasound-negative people were antibody seropositive [18]. It is known that surgical operation of CE is different from that of general cysts. The spillage of viable protoscoleces during the surgery can lead to post-operative reoccurrence of CE while the release of antigenic hydatid fluid into bloodstream can cause anaphylactic shock and even death. Thus, it is important to improve the accurate diagnosis rate before operation by the combined use of different methods in the diagnosis of human CE.

## Conclusions

This is the first retrospective analysis of human CE cases in Heilongjiang Province in the recent ten years. We described the clinical characteristics of CE cases. CT appeared to be the most effective diagnosis method for hepatic CE. Combined with previous reports that dogs and sheep as well as pigs were infected with *E. granulosus* in our investigated areas, ecological environments were inferred to be a main factor leading to the increasing human CE cases. Thus, it is necessary to treat dogs with anticestodal drugs regularly in controlling the spread of CE in humans and animals in Heilongjiang Province.

## References

[1] Tünger Ö (2013). Epidemiology of cystic echinococcosis in the world. Turkiye Parazitol Derg.

[2] Lahmar S, Rebaï W, Boufana BS, Craig PS, Ksantini R, Daghfous A (2009). Cystic echinococcosis in Tunisia: analysis of hydatid cysts that have been surgically removed from patients. Ann Trop Med Parasitol.

[3] Alvarez Rojas CA, Romig T, Lightowlers MW (2014). Echinococcus granulosus sensu lato genotypes infecting humans–review of current knowledge. Int J Parasitol.

[4] Grosso G, Gruttadauria S, Biondi A, Marventano S, Mistretta A (2012). Worldwide epidemiology of liver hydatidosis including the Mediterranean area. World J Gastroenterol.

[5] Zhang T, Yang D, Zeng Z, Zhao W, Liu A, Piao D (2014). Genetic characterization of human-derived hydatid cysts of Echinococcus granulosus sensu lato in Heilongjiang Province and the first report of G7 genotype of E. canadensis in humans in China. PLoS One.

[6] Mahmud AL, Yan W (2011). The geographic distribution, hazard and prevention strategies of Echinococcosis. Xinjiang Med J.

[7] Qu H, Sun Y, Li B, Liu W, Sun Y, Jiang L (2000). Investigation on the larvae of tapeworm in pigs and the tapeworms in dogs in part of cities and counties of Heilongjiang Province, China. Chin J Vet Sci Technol.

[8] Zhang H, Ding Y, Qiao Y (2002). Investigation of parasites in sheep from Shuangcheng Town in Heilongjiang Province, China. Chin J Vet Sci Technol.

[9] Aksu M, Sevimli FK, Ibiloğlu I, Arpacı RB (2013). Cystic echinococcosis in the Mersin province (119 cases). Turkiye Parazitol Derg.

[10] Hajipirloo HM, Bozorgomid A, Alinia T, Tappeh KH, Mahmodlou R (2013). Human cystic echinococcosis in west azerbaijan, northwest iran: a retrospective hospital based survey from 2000 to 2009. Iran J Parasitol.

[11] Xu GR, Zhang LJ, Zeng G (2013). Epidemic analysis of echinococcosis in Ganzi Tibetan Autonomous Prefecture of Sichuan Province from 2006 to 2011. Chin J Parasitol Parasit Dis.

[12] Al-Qaoud KM, Craig PS, Abdel-Hafez SK (2003). Retrospective surgical incidence and case distribution of cystic echinococcosis in Jordan between 1994 and 2000. Acta Trop.

[13] Torgerson PR, Karaeva RR, Corkeri N, Abdyjaparov TA, Kuttubaev OT, Shaikenov BS (2003). Human cystic echinococcosis in Kyrgystan: an epidemiological study. Acta Trop.

[14] Cappello E, Cacopardo B, Caltabiano E, Li Volsi S, Chiara R, Sapienza M (2013). Epidemiology and clinical features of cystic hydatidosis in Western Sicily: a ten-year review. World J Gastroenterol.

[15] Brundu D, Piseddu T, Stegel G, Masu G, Ledda S, Masala G (2014). Retrospective study of human cystic echinococcosis in Italy based on the analysis of hospital discharge records between 2001 and 2012. Acta Trop.

[16] Aaty HE, Abdel-Hameed DM, Alam-Eldin YH, El-Shennawy SF, Aminou HA, Makled SS (2012). Molecular genotyping of *Echinococcus granulosus* in animal and human isolates from Egypt. Acta Trop.

[17] Wahlers K, Menezes CN, Wong M, Mogoye B, Frean J, Romig T (2011). Human cystic echinococcosis in South Africa. Acta Trop.

[18] Shambesh MA, Craig PS, Macpherson CN, Rogan MT, Gusbi AM, Echtuish EF (1999). An extensive ultrasound and serologic study to investigate the prevalence of human cystic echinococcosis in northern Libya. Am J Trop Med Hyg.

[19] Mıman O, Atambay M, Aydin NE, Daldal N (2010). The clinical, serological and morphological analysis of 91 patients with cystic echinococcosis following surgery. Turkiye Parazitol Derg.

[20] Ibrahim MM (2010). Study of cystic echinococcosis in slaughtered animals in Al Baha region, Saudi Arabia: interaction between some biotic and abiotic factors. Acta Trop.

[21] Meng Y, Pamir, Qiu Y, Li K (2000). CT diagnosis of hepatic hydatid cysts. J Chin Clin Med Imaging.

[22] Xu M, Li Z (1999). Ultrasound diagnosis of hepatic echinococcosis in Turpan area, Xinjiang. J Chin Clin Med Imaging.

[23] Suwan Z (1995). Sonographic findings in hydatid disease of the liver: comparison with other imaging methods. Ann Trop Med Parasitol.

[24] Stojkovic M, Rosenberger K, Kauczor HU, Junghanss T, Hosch W (2012). Diagnosing and staging of cystic echinococcosis: how do CT and MRI perform in comparison to ultrasound ?. PLoS Negl Trop Dis.

